# A new numerical approach to solve Thomas–Fermi model of an atom using bio-inspired heuristics integrated with sequential quadratic programming

**DOI:** 10.1186/s40064-016-3093-5

**Published:** 2016-08-23

**Authors:** Muhammad Asif Zahoor Raja, Aneela Zameer, Aziz Ullah Khan, Abdul Majid Wazwaz

**Affiliations:** 1 Department of Electrical Engineering, COMSATS Institute of Information Technology, Attock, Pakistan; 2 Department of Computer and Information Sciences, Pakistan Institute of Engineering and Applied Sciences (PIEAS), Nilore, Islamabad, 45650 Pakistan; 3 Department of Basic Sciences, Riphah International University, Islamabad, Pakistan; 4 Department of Mathematics, Saint Xavier University, Chicago, IL 60655 USA

**Keywords:** Bio-inspired heuristics, Thomas–Fermi model, Atomic physics, Genetic algorithm, Sequential quadratic programming, Nonlinear singular systems

## Abstract

In this study, a novel bio-inspired computing approach is developed to analyze the dynamics of nonlinear singular Thomas–Fermi equation (TFE) arising in potential and charge density models of an atom by exploiting the strength of finite difference scheme (FDS) for discretization and optimization through genetic algorithms (GAs) hybrid with sequential quadratic programming. The FDS procedures are used to transform the TFE differential equations into a system of nonlinear equations. A fitness function is constructed based on the residual error of constituent equations in the mean square sense and is formulated as the minimization problem. Optimization of parameters for the system is carried out with GAs, used as a tool for viable global search integrated with SQP algorithm for rapid refinement of the results. The design scheme is applied to solve TFE for five different scenarios by taking various step sizes and different input intervals. Comparison of the proposed results with the state of the art numerical and analytical solutions reveals that the worth of our scheme in terms of accuracy and convergence. The reliability and effectiveness of the proposed scheme are validated through consistently getting optimal values of statistical performance indices calculated for a sufficiently large number of independent runs to establish its significance.

## Background

The aim of this study is to solve the Thomas–Fermi model of an atom (Fermi 1927; Thomas 1927) represented through nonlinear singular Thomas–Fermi equation (TFE) by exploiting the strength of artificial intelligence algorithms. The fundamental form of TFE is written as:

1 $$y''(t) - t^{ - 1/2} \left( {y(t)} \right)^{3/2} = 0,\quad \text{Re} \,\,t \in [0,\,\,\infty ],$$

along with the associated boundary conditions as:

2 $$y(0) = 1,\quad y(\infty ) = 0,$$

For practical analysis of the dynamics of TFE, most of the studies are conducted for restricted inputs instead of unbounded domain.

The Thomas–Fermi system (1) is one of the simplest approaches to the study of the potential and charge densities in a variety of models, for example, atoms (Banerjee et al. 1974; Coulson and March 1950; March 1957, 1983; March and Tomishina 1979), molecules (Banerjee et al. 1974; March 1952), atoms in strong magnetic fields (Banerjee et al. 1974; March and Tomishina 1979; March 1983), metals and crystals (Umeda and Tomishina 1955) and dense plasmas (Ying and Kalman 1989). The overview, importance and applications of classical numerical approaches for TFE can be seen (Kirzhnits 1957; Bush and Caldwell 1931). The research community has shown great interest in the accurate and reliable calculation of the solution for the TFE including sinc-collocation method (Parand et al. 2013a), Laguerre pseudospectral approximation (Liu and Zhu 2015), Chebyshev pseudospectral method (Kılıçman et al. 2014), rational Chebyshev pseudospectral approach (Parand and Shahini 2009), hermite collocation method (Bayatbabolghani and Parand 2014), rational approximation (Fernández 2011), Homotopy Analysis Method (HAM) (Yao 2008) improved HAM (Zhao et al. 2012), rational Bessel functions collocation method (Parand et al. 2016a), rational Euler functions based methods (Parand et al. 2016b), methods based on Jacobi rational functions with Gauss quadrature formula (Bhrawy and El-Soubhy 2015) and Padé–Hankel method (Amore et al. 2014), Beside these there are many other studies for solving Thomas–Fermi models, see (Fernández 2008; Liao 2003a; Filobello-Nino et al. 2015; Dahmani and Anber 2015; Feng et al. 2015) and the references therein.

After a profound study of the literature regarding Thomas–Fermi equation (TFE) it is observed that only deterministic solvers are applied to analyze its dynamics and no one yet applied the stochastic solvers. Recently, stochastic numerical techniques based on artificial intelligence techniques are effectively used to calculate the accurate solutions for initial and boundary value problems (BVPs) of differential equations involving both integer and fractional derivatives (Parand et al. 2013b; Arqub and Abo-Hammour 2014; Abo-Hammour et al. 2013; Abu Arqub et al. 2012, 2014). For instance, few potential application of stochastic solvers are solution of nonlinear Van-der-Pol oscillatory systems (Khan et al. 2015b), inverse Kinematics problem (Momani et al. 2016), problem arising in Electromagnetic theory (Khan et al. 2015a), nonlinear singular systems (Abo-Hammour et al. 2014a), plasma physics problems (Raja 2014a), nonlinear Navier–Stokes problems (Abo-Hammour et al. 2014b), nanotechnology problems involving carbon nanotubes (Raja et al. 2016a), magnetohydrodynamic problems (Raja et al. 2015a), fuel ignition model of combustion theory (Raja 2014b), fluid dynamics problem of thin film flow (Raja et al. 2016d), mathematical models of electrically conducting solids (Raja et al. 2016b), Schrödinger equation for the hydrogen atom (Caetano et al. 2011), nonlinear Jeffery–Hamel flow in the presence of high magnetic field (Raja and Samar 2014), and strong nonlinear systems based on Painlevé, Bratu, Emden–Fowler, Riccati, Bagley–Torvik, Troesch’s, Lane–Emden, Flierl–Petviashivili and pantograph models (see Mall and Chakraverty 2013, 2014, 2015; Raja et al. 2014, 2015b, c, 2016c) and references therein). Authors motivated from these studies to carry out exploration and exploitation in the field on stochastic numerical solvers to solve governing model of nonlinear TFE based on discritization with finite difference scheme and trained through bio-inspired computing integrated with sequential quadratic programming procedures.

The most incorporated stochastic solvers are normally based on bio-inspired computational heuristics through Genetic Algorithms (GAs), a kind of effective global search methodology. GAs are used extensively to solve variety of the problems arising in various applications in physical sciences (Homayouni et al. 2014; Chiroma et al. 2014, 2015; Toledo et al. 2014; Chiroma et al. 2016) which motivates the authors to exploit the strength of these techniques to study the dynamics of nonlinear singular TFE. The advantages of these methodologies are reflected through simplicity of the concept, ease in implementation processes, wider applicability, avoid divergence, stability, robustness and reliability, which make them impressive to be exploited for challenging models of mathematical physics like TFE. With the advent of modern computer architectures based on signal processing platform, an immense increase in computational power of the machines is achieved which gives a rebirth to population based meta-heuristic methodologies to be used for stiff problems of mathematical physics. The significance of the present research is a step forward in designing the machines learning algorithms for providing the solution of highly nonlinear and singular system for Thomas–Fermi model of an atom given in the form of boundary value problem of TFE for unbounded domain.

The rest of the paper is organized as follows: in “Methods” section, the proposed design methodology based on discretization of differential equation into system of difference equations by using a finite difference scheme is provided along with the optimization procedure for solving system of nonlinear equations; in “Numerical experimentation and results” section, the results of numerical simulations for different cases of TFE are presented in a number of graphs and numerical illustrations; conclusions are listed in the last section along with few suggested research directions.

## Methods

Proposed methodology for Thomas–Fermi Eq. (1) is presented here that consists of two parts. First part is the formulation of optimization problem with the construction of overall individual residual error with the help of finite different schemes satisfying the constrained boundary conditions, while is the second part, a hybrid computing framework based on Genetic Algorithms (GAs) supported with Sequential Quadratic Programming (SQP) is exploited for minimization of the overall residual error function.

### Discretization through finite difference scheme

The simplest and effective technique for solving differential equation is based on finite difference schemes which are used for approximation of derivative terms in the system by using difference quotients.

To obtain the approximate solution of the Thomas–Fermi equation on equally distributed mesh points in the finite interval $t \in [0,\,\,T]$, one can proceed by taking $t_{i} = ih,\quad i = 0,1, \ldots ,N$ for h = 1/N, hence the approximated equation is given for interior mesh points, $t_{i} ,\,\,\,i = 1,2, \ldots ,N - 1$ as:

3 $$y''(t_{i} ) = F(y(t_{i} )),\quad t_{1} \le t_{i} \le t_{N - 1} ,$$

while the boundary conditions are given as:

4 $$y(t_{0} ) = \beta_{1} ,\,\,\,\,y(t_{N} ) = \beta_{2} .$$

Here $F(y(t_{i} )) = \sqrt {{{\left( {y(t_{i} )} \right)^{3} } \mathord{\left/ {\vphantom {{\left( {y(t_{i} )} \right)^{3} } t}} \right. \kern-0pt} t}_{i} } ,\,\,\,\beta_{1} = 1,$ and $\,\beta_{2} = 0$ for Thomas–Fermi equation.

The difference quotients approximation formulates based on 5 interior mesh points is used to closely approximate $y''(t_{i} ),\,\,\quad i = 1,2, \ldots ,N - 1$, by taking small step size *h* with the error on the order of 0 $(h^{3} )$ and are written mathematically for forward Δ, central Π and backward $\nabla$ differences operators, respectively, as:

5 $$y^{\prime\prime}(t_{1} ) = \frac{1}{{h^{2} }}\Delta \left( {y(t_{0} ),y(t_{4} )} \right),$$

6 $$y^{\prime\prime}(t_{i} ) \approx \frac{1}{{h^{2} }}\varPi (y(t_{i - 2} ),y(t_{i + 2} )),\quad i = 2,3, \ldots ,N - 2,$$

7 $$y^{\prime\prime}(t_{N - 1} ) \approx \frac{1}{{h^{2} }}{\nabla }\left( {y(t_{N - 4} ),y(t_{N} )} \right),$$

where forward Δ, central Π and backward ${\nabla }$ differences are defined as:

8 $$\Delta \left( {y(t_{0} ),\,\,y(t_{4} )} \right) = \left( {\frac{11}{12}y(t_{0} ) - \frac{5}{3}y(t_{1} ) + \frac{1}{2}y(t_{2} ) + \frac{1}{3}y(t_{3} ) - \frac{1}{12}y(t_{4} )} \right),$$

9 $$\Pi \left( {y(t_{i - 2} ),y(t_{i + 2} )} \right) = \left( { - \frac{1}{12}y(t_{i - 2} ) + \frac{4}{3}y(t_{i - 1} ) - \frac{5}{2}y(t_{i} ) + \frac{4}{3}y(t_{i + 1} ) - \frac{1}{12}y(t_{i + 2} )} \right)$$

10 $${\nabla }\left( {y(t_{N - 4} ),y(t_{N} )} \right) = \left( { - \frac{1}{12}y(t_{N - 4} ) + \frac{1}{3}y(t_{N - 3} ) + \frac{1}{2}y(t_{N - 2} ) - \frac{5}{3}y(t_{N - 1} ) + \frac{11}{12}y(t_{N} )} \right) .$$

The finite difference schemes are used for solving the Thomas–Fermi differential equations by the procedure of discretization.

### Fitness function formulation

Discretization procedure of differential equations converts the equation to the system of algebraic equations which are then solved by construction of fitness function based on individual residual errors of each equation. The necessary details for the construction of fitness function is given here.

The finite difference approximation formulae for $y^{\prime\prime}(t_{i} ),\quad i = 1,2, \ldots ,N - 1$, given in (5), (6) and (7) are used in (3) to transform the Thomas–Fermi equation as:

11 $$\frac{1}{{h^{2} }}\Delta \left( {y(t_{0} ),y(t_{4} )) - F(y(t_{1} )} \right) \approx 0,$$

12 $$\frac{1}{{h^{2} }}\varPi \left( {y(t_{i - 2} ),y(t_{i + 2} )) - F(y(t_{i} )} \right) \approx 0,\quad i = 2,3, \ldots ,N - 2,$$

13 $$\frac{1}{{h^{2} }}\nabla \left( {y(t_{N - 4} ),y(t_{N} )) - F(y(t_{N - 1} )} \right) \approx 0.$$

Equations (11), (12) and (13) are the system of algebraic equations with *N* dependent variables, i.e., $y(t_{0} ),y(t_{1} ),$ …,$y(t_{N} )$. In order to formulate a fitness function the residual errors *R*_*err*_ are defined as:

14 $$R_{err} (1) = \frac{1}{{h^{2} }}\Delta \left( {y(t_{0} ),y(t_{4} )) - F(y(t_{1} )} \right),$$

15 $$R_{err} (i) = \frac{1}{{h^{2} }}\varPi (y(t_{i - 2} ),y(t_{i + 2} )) - F(y(t_{i} )),\quad i = 2,3, \ldots ,N - 2,$$

16 $$R_{err} (N - 1) = \frac{1}{{h^{2} }}\nabla \left( {y(t_{N - 4} ),y(t_{N} )) - F(y(t_{N - 1} )} \right),$$

The overall individual residual function *O*_*R*_ is defined, similar to $l_{2}$ the norm of the residuals of all nodes and it is given mathematically as:

17 $$O_{R} = \sqrt {\left( {R_{err} (1)} \right)^{2} + \sum\limits_{i = 2}^{N - 2} {\left( {R_{err} (i)} \right)^{2} + \left( {R_{err} (N - 1)} \right)^{2} ,} }$$

Now the requirement is to minimize the fitness function *O*_*R*_ for which the value of individual residual errors for each equation decreases. Consequently, the desired results or optimized solutions of the problem are achieved when *O*_*R*_ approaches zero.

### Learning methodology

Residual error function (17) is minimized through hybrid computing approach consisting of GAs integrated with SQP method, i.e., GA-SQP algorithms.

First real application of GAs has been given by Holland (Holland 1975) in early 70’s of the last century and afterwards GAs is used as one of the premier derivative free solver for both constrained and non-constrained optimization problems. GAs belongs to the class of global search methods formulated through mathematical modeling of natural genetic mechanism. The standard operation of GAs is based on its reproduction operators, which are selection, crossover, and mutation. GAs are applied effectively as a good optimization mechanism in diverse fields such as electronics, optics, electromagnetism, controls, digital communication, robotics, astrophysics, chemical industry, materials, signal processing, nuclear power systems, bioinformatics, economics, and financial mathematics etc. (see Haupt and Haupt 2004; Kumar et al. 2010; Dasgupta and Michalewicz 2013; Grefenstette 2013, and references therein). Few recently reported potential applications of GAs are optimization in orbital maneuvers (dos Santos and da Silva Formiga 2015), overlapping community detection in complex networks (Yuxin et al. 2015), formulation of a public bicycle-sharing system (Askari and Bashiri 2015), preemptive identical parallel machines scheduling problem (Aalaei et al. 2015) and spacecraft guidance and control system (Shirazi and Mazinan 2015).

**Fig. 1:**
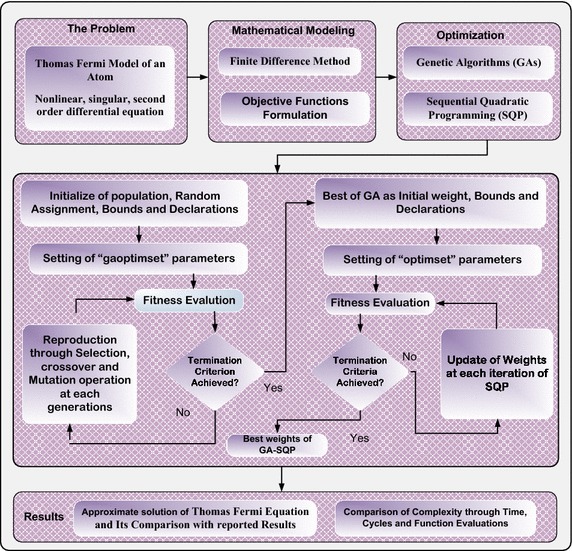
Graphical abstract of proposed methodology for solving Thomas–Fermi equation

The GAs is implemented through built-in functions available in the MATLAB optimization toolbox and for effective optimization. GAs are hybridized with SQP for rapid local search. In this manner, a hybrid meta-heuristic optimization mechanism is designed for training of weights of ANNs based on GAs integrated with SQP to solve Thomas–Fermi equation. Detailed workflow of the proposed design scheme, in terms of the problem, modeling, optimization procedures and comparison, are shown in Fig. 1. Optimization of the fitness function given in Eq. (17) is carried out with the help of GA-SQP, which are implemented through built-in routines of GAs and FMINCON with algorithm SQP available in the Matlab optimization toolbox. The parameter settings applied for GAs and SQP are given in Table 1. These settings are made with care, after a lot of experimentation. A slight variation in these settings may result in pre-mature convergence of the algorithms.

**Table 1:** Parameters settings used for the genetic algorithms (GAs) and sequential quadratic programming (SQP)

Methods	Parameters	Settings	Parameters	Settings
GAs	Population creation	Uniform	Individual size	9, 19, 49
	‘PopulationSize’	200	‘Generation’	400
	Selection function	Stochastic uniform	FunctionTolerance ‘TolFun’	10^−24^
	Initial population range	[−0.5, 0.5]	ConstraintTolerance ‘TolCon’	10^−24^
	Crossover function	@crossoverheuristic	Lower bounds for all entries	−5
	Mutation function	@mutationadaptivefeasible	Upper bounds for all entries	5
	‘EliteCount’	4	‘StallGenLimit’	100
	‘FitnessLimit’	10^−15^	Other	Defaults
SQP	Initial weights	Global best of GAs	Bounds [lower, upper]	[−5, 5]
	Algorithm	‘SQP’	Finite difference	‘Central’
	Maximum iterations	1000	‘TolX’	10^−15^
	Function counts	150,000	‘TolCon’	10^−24^
	Other	Defaults	‘TolFun’	10^−24^

The detailed description of the procedural steps of proposed GA-SQP algorithm is given as follows: Firstly, the initial chromosome for GAs are created with bounded real values randomly having genes or elements equal to the number of unknown variables in the residual error function. These chromosomes formulate an initial population ***P*** for the algorithm. Mathematically the population P based on chromosomes is given as:

$$\begin{aligned} \varvec{P} &= \left[ {\varvec{c}_{ 1} ,\varvec{c}_{ 2} , \ldots ,\varvec{c}_{M} } \right]^{T} ,\, \hfill \\ \varvec{c}_{i} &= [c_{1} ,\,c_{2} , \ldots ,c_{N} ]_{i} = [y_{1} ,\,y_{2} ,\, \ldots ,y_{N} ]_{i} , \hfill \\ \end{aligned}$$

where *M* is the total number of chromosomes in the population, while each chromosome has M elements or genes which represents the discritization points of finite difference scheme. The rest of parameters are set for GA as listed in Table 1. In second step, the value of fitness $O_{R}$ is determined for each chromosome ***c*** of the population *P* using Eq. (17) and its constituent parts given in Eqs. (14–16). Each chromosome ***c*** of the population ***P*** with a minimum value of fitness $O_{R}$ is ranked high and vice versa. Ranking each chromosome of the population accordingly. Thirdly, algorithm stops its execution in case of fulfillments of the fitness limit; total number of generations/cycles executed, tolerance limits are attained such as function tolerance (TolFun) and nonlinear constraint tolerance (TolCon). If termination criteria satisfied just go SQP algorithm otherwise reproduced population using crossover, mutation and selection operations by invoking the built-in functions for these genetic operators, as listed in Table 1. Repeat the procedure accordingly. The rapid refinement of the results is carried out using SQP algorithm by using ‘fmincon’ routine with initial weights which are the global best solution of GAs. Initial declarations, setting and boundes for the algorithm are listed in Table 1. Determined the value of fitness $O_{R}$, as given in the Eq. (17) for each updated weights vector by SQP procedure and terminate the cyclic updating of weights if execution of total number of iterations, fitness, tolerances limits are achieved and refined design parameter are obtained. Finally, store the final optimized weights of both GA and GA-SQP algorithms along with their fitness, time consumed, generations executed, and functions evaluated.

## Numerical experimentation and results

The results of numerical experiments are presented in this section for solving Thomas–Fermi equation by taking five scenarios based on the size of input interval, while in each scenario three cases are taken with different values of the step size parameter. The scenarios with different cases are given as follows:

### *Scenario 1*

Study the dynamics of TFE for input span $t \in [0,\,\,1]$ with cases 1, 2 and 3 based on step size parameter *h* = 0.1, 0.05, and 0.02, respectively. Mathematically the model Eq. (1) for this scenario along with related boundary condition is given as:

18 $$\begin{aligned} & y''(t) - t^{ - 1/2} \left( {y(t)} \right)^{3/2} = 0, \quad \text{Re} \,t \in [0,\,\,1], \hfill \\ & y(0) = 1,\,\,\,\,\hat{y}(1) = 1 \hfill \\ \end{aligned}$$

### *Scenario 2*

In this scenario, TFE for input span $t \in [0,\,\,5]$ with cases 1, 2 and 3 based on step size parameter *h* = 0.5, 0.25, and 0.1, respectively is taken and mathematically is given as:

19 $$\begin{aligned} & y''(t) - t^{ - 1/2} \left( {y(t)} \right)^{3/2} = 0, \quad \text{Re} \,t \in [0,\,\,5], \hfill \\ & y(0) = 1,\,\,\,\,\hat{y}(5) = 0 \hfill \\ \end{aligned}$$

### *Scenario 3*

TFE (1) for input span $t \in [0,\,\,25]$ with cases 1, 2 and 3 based on step size parameter *h* = 2.5, 1.25, and 0.5, respectively is taken in this scenario and it is written as:

20 $$\begin{aligned} & y''(t) - t^{ - 1/2} \left( {y(t)} \right)^{3/2} = 0, \quad \text{Re} \,t \in [0,\,\,25], \hfill \\ & y(0) = 1,\,\,\,\,\hat{y}(25) = 0 \hfill \\ \end{aligned}$$

### *Scenario 4*

Study the dynamics of Thomas–Fermi model for relatively larger input span $t \in [0,\,\,50]$ with cases 1, 2 and 3 based on step size parameter *h* = 5.0, 2.5, and 1.0, respectively. Mathematically the model Eq. (1) for this scenario is given as:

21 $$\begin{aligned} & y''(t) - t^{ - 1/2} \left( {y(t)} \right)^{3/2} = 0, \quad \text{Re} \,t \in [0,\,\,50], \hfill \\ &y(0) = 1,\,\,\,\,\hat{y}(50) = 0 \hfill \\ \end{aligned}$$

### *Scenario 5*

Solution of TFE is analyzed for larger input span $t \in [0,\,\,100]$ with cases 1, 2 and 3 based on step size parameter *h* = 10.0, 5.0, and 2.0, respectively. Mathematically the model Eq. (1) for this scenario is written as:

22 $$\begin{aligned} & y''(t) - t^{ - 1/2} \left( {y(t)} \right)^{3/2} = 0, \quad \text{Re} \,t \in [0,\,\,100], \hfill \\ & y(0) = 1,\,\,\,\,\hat{y}(100) = 0 \hfill \\ \end{aligned}$$

Design methodology is applied to obtain the solution of Thomas–Fermi equation for all three cases of each scenario as per procedure given in the last section. Fitness function as given in Eq. (17) for inputs $t \in [0,\,\,1]$ with step size *h* = 0.1, 0.05, 0.02 i.e., N = 10, 20, 50, for cases 1, 2 and 3, are formulated, respectively as:

23 $$O_{R} = \sqrt {\left( {R_{err} (1)} \right)^{2} + \sum\limits_{i = 2}^{8} {\left( {R_{err} (i)} \right)^{2} + \left( {R_{err} (9)} \right)^{2} ,} }$$

24 $$O_{R} = \sqrt {\left( {R_{err} (1)} \right)^{2} + \sum\limits_{i = 2}^{18} {\left( {R_{err} (i)} \right)^{2} + \left( {R_{err} (19)} \right)^{2} ,} }$$

25 $$O_{R} = \sqrt {\left( {R_{err} (1)} \right)^{2} + \sum\limits_{i = 2}^{48} {\left( {R_{err} (i)} \right)^{2} + \left( {R_{err} (49)} \right)^{2} ,} }$$

Accordingly, fitness functions $O_{R}$ are developed for all three cases of scenarios 2–5.

**Fig. 2:**
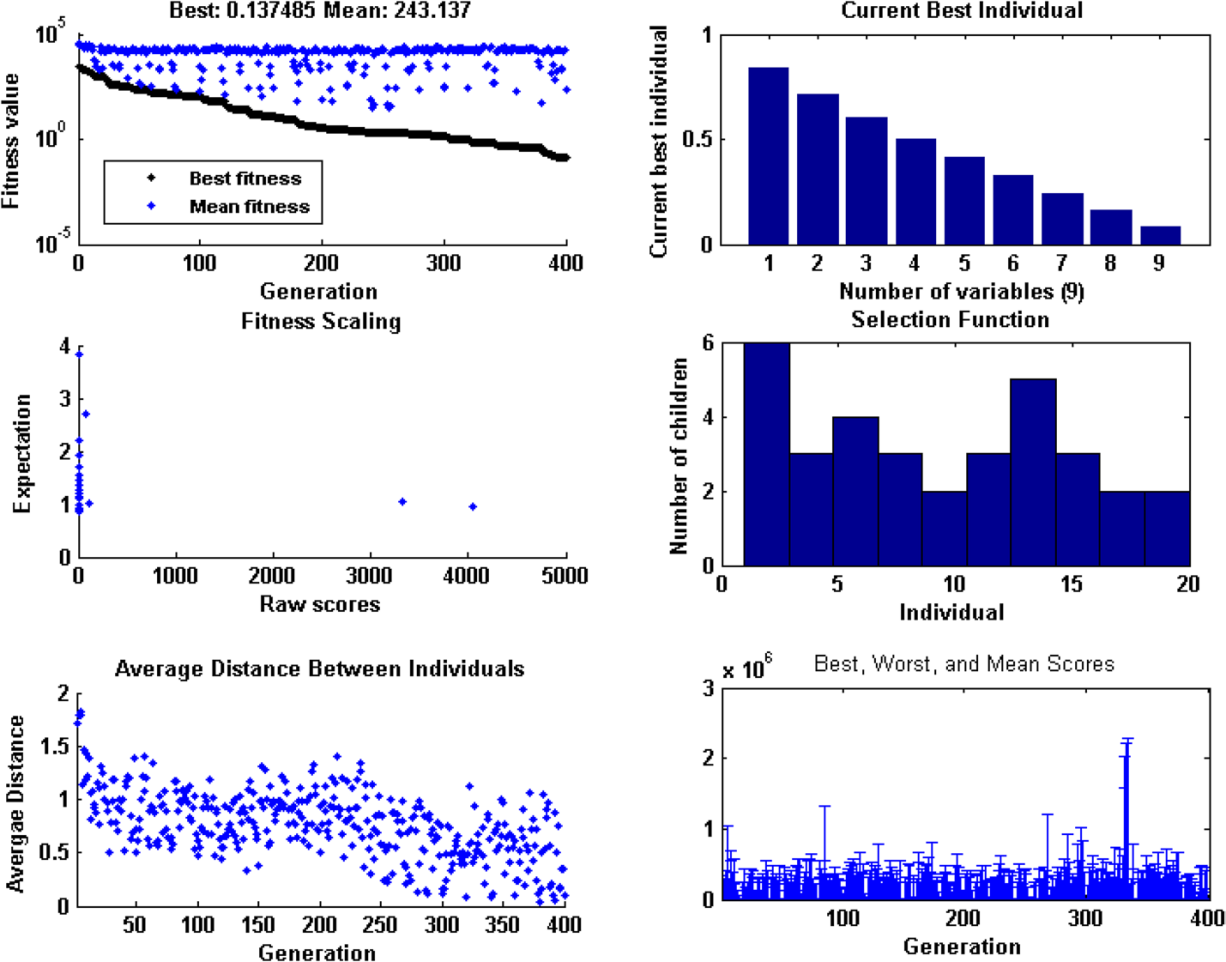
Optimization output plots of GAs to solve scenario 1: case 1 of Thomas–Fermi equation

**Fig. 3:**
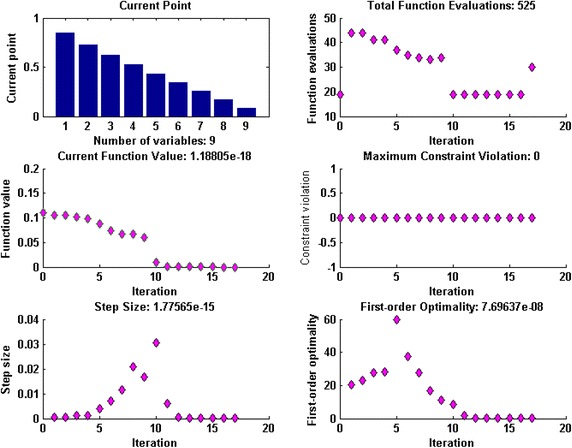
Optimization output plots of GA-SQP to solve scenario 1: case 1 of Thomas–Fermi equation

**Fig. 4:**
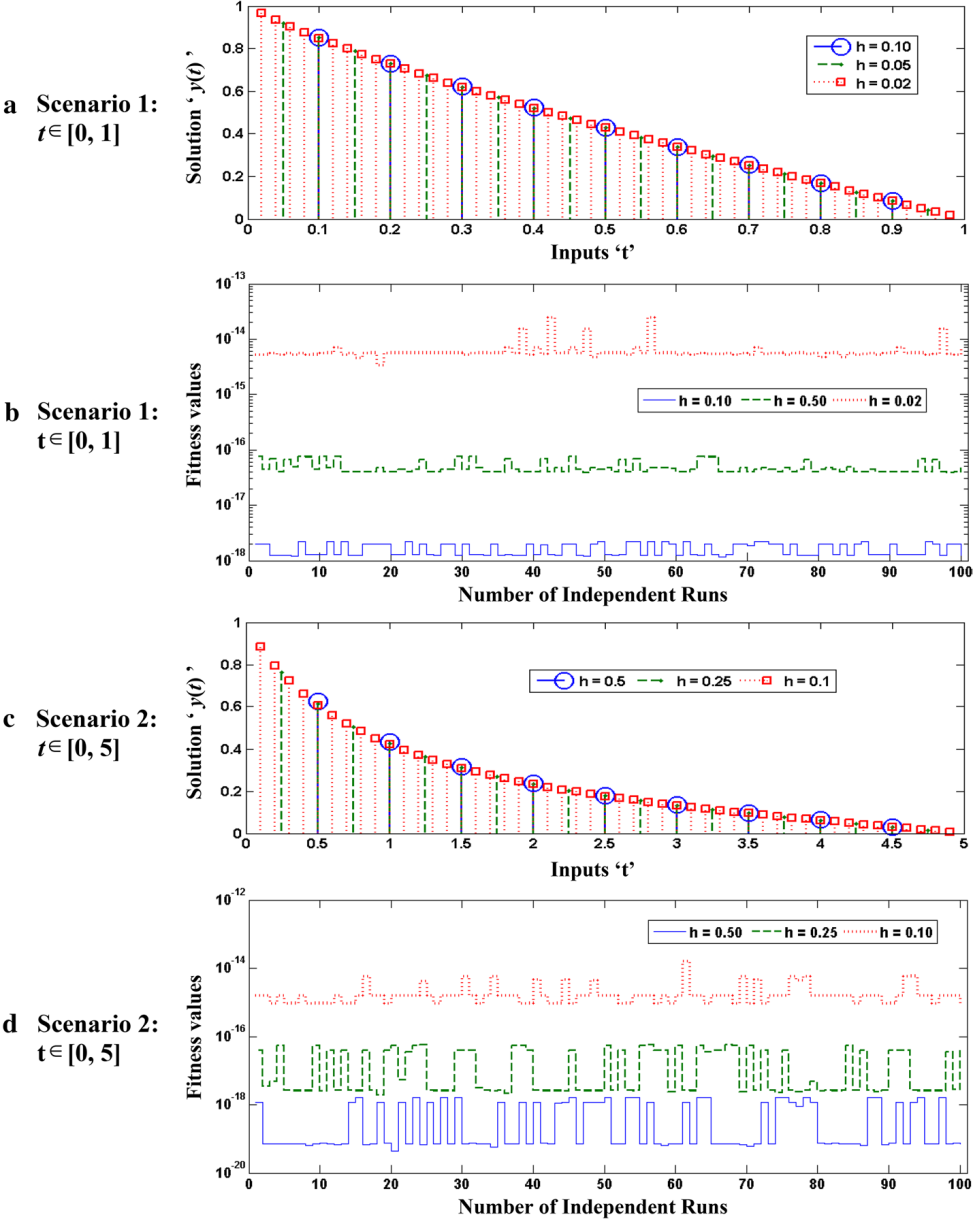
Results for all three cases of scenario 1 and 2 of Thomas–Fermi equation, **a**, **c** for the proposed approximate solutions while **b**, **d** are for graphical representation of statistical analysis based on 100 runs of the algorithms

**Fig. 5:**
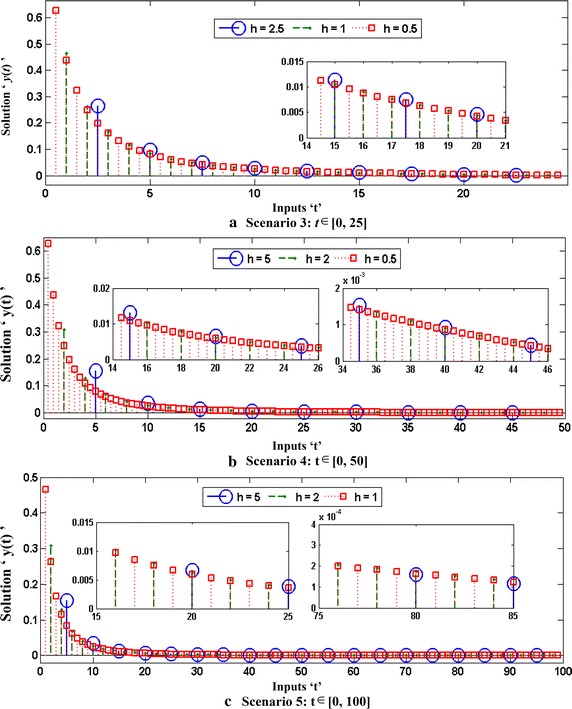
Proposed approximate solutions of Thomas–Fermi equation for all three cases of scenarios 3, 4 and 5

**Fig. 6:**
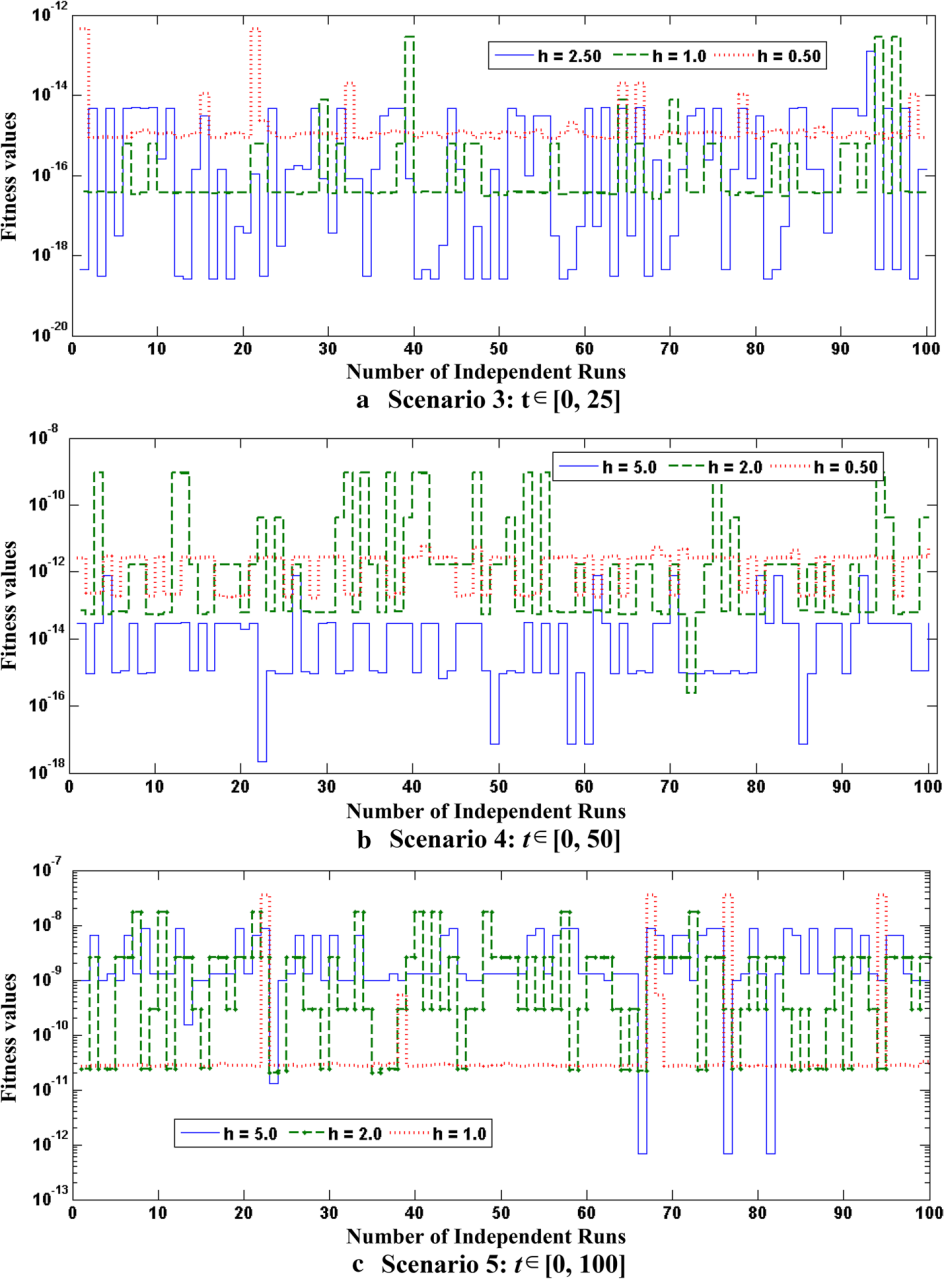
Graphical representation of statistical analysis for all three cases of scenarios 3, 4, and 5 of Thomas–Fermi equation based on 100 runs of the algorithms

Optimization of fitness function $O_{R}$ for each case of all five scenarios are carried out with the help of a hybrid computing approach based on GA-SQP in case of 100 independent runs using parameter settings as listed in Tables 1. Optimization output plots for the fitness function $O_{R}$ by GAs in terms of fitness values, current best chromosome, fitness scaling, selection, average distance between the individuals, best, mean and worst scores are shown in Fig. 2 for the case 1 of scenario 1. Accordingly, for the same case optimization plots of SQP algorithms in terms of current best point, function counts, learning curves, constraints violation, step size parameter, and first-order optimality, are given in Fig. 3. Similarly the optimization outputs for other cases are determined and results obtained with one of the runs of GA-SQP algorithms for each case of scenarios 1 and 2, are presented in Fig. 4, along with the values of fitness plotted against 100 independent runs of the GA-SQP algorithm. The fitness values are plotted on a semi-log scale in order to observe the small variation in the results. Accordingly, results of proposed solutions for all three cases of 3, 4 and 5 scenarios are given in Fig. 5, while result of statistical analysis are plotted in Fig. 6 for each scenario. In case of Fig. 4a the plots of all three cases based on values of step sizes *h* = 0.1, 0.05 and 0.02 are consistently overlapping. In case of Fig. 4b, it is seen that with the decrease in step size, the value of fitness also decreases, which is due to the fact that with the decreased step size, the discretization of the system using the finite difference scheme based on increased number of nonlinear equations. Consequently, the system becomes stiff with a decrease in step size and hence the solution is determined with less accuracy, generally by all methods. The same inferences and trend have been observed for scenarios 2–5 but the level of matching the results degraded because with more number of mesh points, i.e., for smaller values of *h*, the smooth results are obtained which is not possible for few mesh points. Additionally, it seems that small variations in the results are observed in each case of all five scenarios in the study, but closely seen reveals that for larger input span the variation in the results is rather more frequent.

The statistical indicator based on the minimum (MIN), mean and standard deviation (STD) values are calculated for 100 independent runs of the proposed scheme for each case of all five scenarios of the problem and results are given in Table 2 for five inputs. While in Tables 3 and 4 the statistical indices are given for more intermediate inputs to analyze dynamics of the problem for few selected cases and scenarios of TFE. From the values presented in these tables, no noticeable difference is apparently observed between the MIN and mean values because of very small values of STD for each case of all five scenarios. Additionally, it is seen that from smaller input span t ∈ [0, 1] to larger inputs t ∈ [0, 100], the values of STD degraded but still remains of the order of 10^−09^, which established the consistency of the proposed methodology for solving TFE.

**Table 2:** Results of statistical indices for each case of all five scenarios of Thomas–Fermi equation

Scenario	*t*	Case 1			*t*	Case 2			*t*	Case 3		
		Min	Mean	SD		Min	Mean	SD		Min	Mean	SD
1	0.1	0.851971	0.851971	4.52E−12	0.05	0.920142	0.920142	6.08E−12	0.02	0.96585	0.96585	3.51E−11
	0.3	0.62099	0.62099	8.72E−12	0.25	0.672508	0.672508	2.36E−11	0.26	0.661314	0.661314	1.27E−10
	0.5	0.428691	0.428691	9.18E−12	0.45	0.473805	0.473805	3.37E−11	0.5	0.427647	0.427647	4.20E−10
	0.7	0.253065	0.253065	6.81E−12	0.65	0.295554	0.295554	3.19E−11	0.74	0.21841	0.21841	4.27E−10
	0.9	0.083907	0.083907	2.47E−12	0.85	0.125711	0.125711	1.72E−11	0.98	0.016734	0.016734	4.78E−11
2	0.5	0.626064	0.626064	4.02E−11	0.25	0.763272	0.763272	8.48E−11	0.1	0.883944	0.883944	1.37E−10
	1.5	0.315987	0.315987	1.15E−10	1.25	0.36109	0.36109	4.17E−10	1.3	0.34772	0.34772	1.80E−09
	2.5	0.17976	0.17976	1.67E−10	2.25	0.20325	0.20325	6.86E−10	2.5	0.175894	0.175894	3.02E−09
	3.5	0.096649	0.096649	1.61E−10	3.25	0.113205	0.113205	7.48E−10	3.7	0.081102	0.081102	2.77E−09
	4.5	0.031067	0.031067	7.40E−11	4.25	0.046107	0.046107	4.65E−10	4.9	0.006074	0.006074	3.14E−10
3	2.5	0.264695	0.264695	2.36E−09	1	0.465685	0.465685	9.48E−10	0.5	0.627672	0.627672	3.37E−10
	7.5	0.048052	0.048052	2.12E−08	6	0.062298	0.062298	1.82E−08	6.5	0.052959	0.052959	1.65E−08
	12.5	0.017323	0.017323	5.87E−08	11	0.020782	0.020782	6.17E−08	12.5	0.015596	0.015596	5.91E−08
	17.5	0.007506	0.007507	8.53E−08	16	0.00898	0.00898	1.07E−07	18.5	0.005748	0.005748	8.54E−08
	22.5	0.002241	0.002241	5.16E−08	21	0.003412	0.003412	9.43E−08	24.5	0.000409	0.000409	1.33E−08
4	5	0.154691	0.154691	7.11E−09	2	0.308917	0.308917	4.73E−08	0.5	0.627672	0.627672	3.08E−10
	15	0.013087	0.013087	1.56E−07	12	0.018663	0.018664	2.66E−06	12.5	0.053001	0.053001	2.05E−08
	25	0.003821	0.003821	5.80E−07	22	0.004943	0.004949	1.26E−05	24.5	0.015904	0.015904	1.07E−07
	35	0.001523	0.001524	9.78E−07	32	0.001918	0.001931	2.60E−05	36.5	0.006928	0.006929	2.94E−07
	45	0.000442	0.000442	6.49E−07	42	0.000699	0.000711	2.52E−05	48.5	0.003631	0.003631	5.66E−07
5	5	0.154691	0.154691	2.88E−07	2	0.308917	0.308917	4.60E−08	1	0.465686	0.465686	1.44E−08
	25	0.003863	0.003908	2.92E−05	26	0.003357	0.003378	2.47E−05	25	0.003554	0.003557	1.67E−05
	45	0.000886	0.001081	0.00011	50	0.000663	0.000766	0.000107	49	0.000679	0.000695	7.55E−05
	65	0.000326	0.000663	0.000169	74	0.000228	0.000385	0.000152	73	0.000229	0.000253	0.000109
	85	0.000117	0.000409	0.000141	98	1.85E−05	4.59E−05	2.65E−05	97	2.55E−05	3.08E−05	2.42E−05

**Table 3:** Results of statistical indices at intermediated inputs in case 1 of all five scenarios of Thomas–Fermi equation

Scenario	Inputs	Statistical Operators			Scenario	Inputs	Statistical Operators		
	t	Min	Mean	SD		t	Min	Mean	SD
1	0.1	0.851971	0.851971	4.52E−12	2	0.5	0.626064	0.626064	4.02E−11
	0.2	0.729282	0.729282	7.13E−12		1	0.432718	0.432718	7.77E−11
	0.3	0.62099	0.62099	8.72E−12		1.5	0.315987	0.315987	1.15E−10
	0.4	0.521817	0.521817	9.35E−12		2	0.237314	0.237314	1.46E−10
	0.5	0.428691	0.428691	9.18E−12		2.5	0.17976	0.17976	1.67E−10
	0.6	0.339585	0.339585	8.31E−12		3	0.134563	0.134563	1.74E−10
	0.7	0.253065	0.253065	6.81E−12		3.5	0.096649	0.096649	1.61E−10
	0.8	0.168091	0.168091	4.80E−12		4	0.062842	0.062842	1.27E−10
	0.9	0.083907	0.083907	2.47E−12		4.5	0.031067	0.031067	7.40E−11
3	2.5	0.264695	0.264695	2.36E−09	4	5	0.154691	0.154691	7.11E−09
	5	0.098161	0.098161	8.86E−09		10	0.035118	0.035118	4.84E−08
	7.5	0.048052	0.048052	2.12E−08		15	0.013087	0.013087	1.56E−07
	10	0.027592	0.027592	3.87E−08		20	0.006575	0.006575	3.39E−07
	12.5	0.017323	0.017323	5.87E−08		25	0.003821	0.003821	5.80E−07
	15	0.011384	0.011384	7.65E−08		30	0.002387	0.002388	8.23E−07
	17.5	0.007506	0.007507	8.53E−08		35	0.001523	0.001524	9.78E−07
	20	0.004642	0.004642	7.66E−08		40	0.000924	0.000925	9.20E−07
	22.5	0.002241	0.002241	5.16E−08		45	0.000442	0.000442	6.49E−07

**Table 4:** Results of statistical operator at intermediated inputs for selected cases and scenarios of Thomas–Fermi equation

Scenario	Cases	*t*	Statistical indices			Scenario	Case	*t*	Statistical indices		
			Min	Mean	SD				Min	Mean	SD
1	1	0.02	0.96585	0.96585	3.51E−11	2	3	0.1	0.883944	0.883944	1.37E−10
		0.08	0.876674	0.876674	6.80E−11			0.4	0.65996	0.65996	5.50E−10
		0.14	0.79854	0.79854	4.98E−11			0.7	0.519596	0.519596	9.70E−10
		0.2	0.727403	0.727403	5.79E−11			1	0.421072	0.421072	1.39E−09
		0.26	0.661314	0.661314	1.27E−10			1.3	0.34772	0.34772	1.80E−09
		0.32	0.59905	0.59905	2.10E−10			1.6	0.290828	0.290828	2.18E−09
		0.38	0.539755	0.539755	2.91E−10			1.9	0.245241	0.245241	2.53E−09
		0.44	0.482787	0.482787	3.63E−10			2.2	0.20767	0.20767	2.81E−09
		0.5	0.427647	0.427647	4.20E−10			2.5	0.175894	0.175894	3.02E−09
		0.56	0.373937	0.373937	4.58E−10			2.8	0.148352	0.148352	3.14E−09
		0.62	0.321333	0.321333	4.73E−10			3.1	0.123906	0.123906	3.15E−09
		0.68	0.269564	0.269564	4.63E−10			3.4	0.101707	0.101707	3.03E−09
		0.74	0.21841	0.21841	4.27E−10			3.7	0.081102	0.081102	2.77E−09
		0.8	0.167686	0.167686	3.66E−10			4	0.061587	0.061587	2.37E−09
		0.86	0.11724	0.11724	2.80E−10			4.3	0.042767	0.042767	1.81E−09
		0.92	0.066953	0.066953	1.72E−10			4.6	0.024337	0.024337	1.12E−09
		0.98	0.016734	0.016734	4.78E−11			4.9	0.006074	0.006074	3.14E−10

Comparative study for the proposed solution of TFE is made with the results of existing techniques based on Rational Chebyshev PseudoSpectral Method (RCPSM) (Parand and Shahini 2009), Homotopy Analysis Method with Transform Approach (HAMTA) (Khan and Xu 2007), Nonlinear Distribution Homotopy Perturbation Method (NDHPM) (Filobello-Nino et al. 2015), and Variational Iterational Method (VIM) (He 2006). Results of the proposed scheme and reported solutions of RCPSM, HAM, NDHPM and VIM are given in Table 5 for inputs *t* ∈ [0, 5]. It can be seen that trend of the proposed results is aligned with the similar patterns of state of the art numerical and analytical solutions. Moreover, comparison of the results is presented on larger inputs span *t* ∈ [0, 100] in Table 6 for the proposed and reference solver based on HAMTA (Khan and Xu 2007), HAM (Liao 2003b) and Chebyshev PseudoSpectral Method (CPSM) (Kılıçman et al. 2014). The similarity of the proposed solutions is observed for a larger input span of TFE from reference standard results.

**Table 5:** Comparison of proposed solution with reported results of state of art numerical and analytical solver in case of scenarios 2 for t ∈ [0, 5]

*t*	Reported solutions				Present results
	RCPSM	HAM	NDHPM	VIM	
0	1	1	1	1	1
0.25	0.755881	0.776191	0.708503	0.68065	0.763272
0.5	0.6067	0.615917	0.570492	0.459456	0.611848
0.75	0.502964	0.50538	0.485018	0.307043	0.504675
1	0.424333	0.423772	0.421167	0.202656	0.424096
1.25	0.363228	0.362935	0.369542	0.131668	0.36109
1.5	0.314661	0.31449	0.326299	0.0838	0.310373
1.75	0.275234	0.275154	0.289316	0.051853	0.268571
2	0.242679	0.242718	0.257254	0.030802	0.233401
2.25	0.215439	0.21563	0.22921	0.017153	0.20325
2.5	0.192406	0.192795	0.20454	0.008491	0.176941
2.75	0.172759	0.173364	0.182755	0.003153	0.153592
3	0.155872	0.156719	0.163464	0	0.132524
3.25	0.141261	0.142371	0.146346	−0.00174	0.113205
3.5	0.128541	0.129937	0.131129	−0.00258	0.095214
3.75	0.117408	0.119108	0.117581	−0.00287	0.07821
4	0.107613	0.109632	0.105505	−0.00285	0.061916
4.25	0.098954	0.101303	0.094726	−0.00264	0.046107
4.5	0.091266	0.09395	0.085097	−0.00235	0.030601
4.75	0.084412	0.087432	0.076486	−0.00204	0.015259
5	0.078278	0.08163	0.068779	−0.00173	0

**Table 6:** Comparison of proposed solution with reported results of state of art numerical and analytical solver in case of inputs *t* ∈ [0, 100]

Inputs *t*	Reported solutions			Present results
	HAMTA	HAM	CPSM	
0.5	0.615917	0.606987	0.605271	0.627672
1	0.423772	0.424008	0.420344	0.436609
1.5	0.31449	0.314778	0.318737	0.323121
2	0.242718	0.243009	0.256011	0.248928
2.5	0.192795	0.192984	0.213705	0.197372
3	0.156719	0.156633	0.183319	0.159988
3.5	0.129937	0.12937	0.160461	0.131997
4	0.109632	0.108404	0.142654	0.110501
4.5	0.09395	0.091948	0.128394	0.093649
5	0.08163	0.078808	0.11672	0.062332
6	0.063816	0.059423	0.098753	0.048199
7	0.051801	0.046098	0.085573	0.038151
8	0.043286	0.036587	0.075495	0.030784
9	0.037002	0.029591	0.067539	0.025244
10	0.032208	0.024314	0.061099	0.011138
15	0.019184	0.010805	0.041371	0.005936
20	0.013494	0.005785	0.031272	0.003554
25	0.010357	0.003474	0.025135	0.000645
50	0.004731	0.000632	0.012687	0.00021
75	0.003052	0.000218	0.008485	2.66E−11
100	0.002251	0.0001	0.006374	0

### Comparative analysis on global performance operators

Comparative analysis of the results is made on the basis of the mean value of fitness to analyze the accuracy and convergence and in case of examining the complexity operator based on mean time, mean generations and mean function counts are incorporated. The mean and STD values of fitness, time, generations and function counts are given in Table 7 for each case of all five scenarios of TFE. It is seen quite apparently that with the increase in length of input span, i.e., moving from scenario 1 to scenario 5, the accuracy of the algorithms decreases due to the fact that for large input span, handling of nonlinearity and singularity associated with TFE is rather more difficult.

**Table 7:** Comparative analysis based on global performance operators for each case of all five scenarios of Thomas–Fermi equation

Scenario	Cases	Fitness		Time		Generations		Function counts	
		Mean	SD	Mean	SD	Mean	SD	Mean	SD
1	1	1.62E−18	3.96E−19	6.161589	0.080307	438.39	2.394839	60,977.77	52.5399
	2	4.88E−17	1.21E−17	10.20638	0.157212	480.48	4.52709	63,510.95	195.6724
	3	6.28E−15	3.18E−15	23.34609	0.940669	625.59	56.40924	83,188.91	5815.303
2	4	5.68E−19	6.54E−19	6.479431	0.068527	434.82	2.844026	60,875.68	55.94963
	5	2.02E−17	2.17E−17	10.11654	0.13137	466.38	3.451775	62,892.43	141.2448
	6	2.07E−15	2.03E−15	22.62986	0.373598	563.98	5.029167	76,831.1	499.5027
3	7	2.88E−15	1.26E−14	6.495513	0.069642	454.65	7.115746	61,220.97	135.6361
	8	8.97E−15	4.91E−14	11.99062	0.182124	494.99	3.208244	64,961.87	161.0217
	9	1.12E−14	6.49E−14	22.47932	0.381859	556.48	4.766211	76,010.69	484.2628
4	10	6.76E−14	1.94E−13	6.470362	0.092306	468.14	4.005098	61,468.95	76.0917
	11	1.23E−10	3.06E−10	12.02846	0.169871	502.29	6.13204	65,274.93	303.6439
	12	2.09E−12	1.43E−12	50.65287	2.698807	705.28	72.3658	12,1735	14711.01
5	13	3.26E−09	3.12E−09	10.06744	0.188208	489.28	9.646353	63,682.09	377.1915
	14	2.69E−09	4.75E−09	22.21883	0.396756	546.91	7.305865	74,967.26	732.745
	15	1.44E−09	6.89E−09	52.35729	3.308367	745.53	70.99686	130,043.8	14,415.26

It is seen from the results presented in Table 7 that the values of mean time for optimization of fitness function by GA-SQP algorithm in case of 11, 21, 51 and 101 input grid points are 6 ± 1, 11 ± 1, 22 ± 1, and 50 ± 1 s. While the number of generations is around 440 ± 10, 480 ± 10, 600 ± 20, and 720 ± 25 for 11, 21, 51 and 101 input grid points and respective values for function counts are around 60,950 ± 60, 63,400 ± 200, 76,500 ± 700 and 125,000 ± 14,000. With an increase in the number of grid points, the complexity of the fitness function increases due to which larger values of complexity operator are obtained.

### Conclusions

Although there has been achieved a number of solutions of Thomas–Fermi Equation but the highly impressive potent outcome of our study are summarized by the following concluding remarks:

- A novel design is presented for solving nonlinear singular Thomas–Fermi equation with the help of finite difference method for discritization of problem and resultant system of nonlinear equations are solved by exploiting the strength of bio-inspired computing technique based on genetic algorithms hybrid with sequential quadratic programming.
- Design scheme is applied to a number of variants of Thomas–Fermi equations by taking different input spans to probe the solution of the problem and it is found that the given scheme is equally reliable and effective for both small and large inputs intervals.
- To draw concrete inferences of the proposed scheme, statistical analysis is performed for a sufficient large number of independent executions of the algorithm and consistently getting similar values of statistical operators with small values of STD establishes the acceptability of the scheme.
- Comparison of the proposed approximate solutions with the reported results of the state of the art numerical and analytical solvers demonstrate that the given scheme is an accurate, viable and preferable alternate platform for studying the dynamics of Thomas–Fermi equation based on artificial intelligence techniques.
- Comparison of the results on the basis of mean values of residual error or fitness function shows that for wider input span the performance of the proposed scheme is slightly degraded but still achieving the values of fitness of the order 10^−09^.
- Computational complexity of the proposed scheme is examined through an average values of time, generations and function counts being consumed by GA-SQP algorithm for optimization of fitness functions and it is found that with increase in the number of grid points, the values of complexity operators are on the higher side because in these cases more computational drive is required for solving the decretization model of the equation.
- Beside the consistent accuracy and convergence, other perks of the proposed scheme are simplicity of the concept, easy implementation and a good alternate avenue to be exploited for solving the nonlinear and singular problems for which the conventional methodologies fail.

In future, present research may prove to be a beacon for researchers working in the domain of application of artificial intelligence techniques to stiff problems arising in physical models of practical importance.
